# Exploring the Functional Complementation between Grp94 and Hsp90

**DOI:** 10.1371/journal.pone.0166271

**Published:** 2016-11-08

**Authors:** Kevin A. Maharaj, Nanette L. S. Que, Feng Hong, John D. Huck, Sabrina K. Gill, Shuang Wu, Zihai Li, Daniel T. Gewirth

**Affiliations:** 1 Hauptman-Woodward Medical Research Institute, Buffalo, New York, United States of America; 2 Department of Structural Biology, University at Buffalo, Buffalo, New York, United States of America; 3 Department of Microbiology and Immunology, Hollings Cancer Center, Medical University of South Carolina, Charleston, South Carolina, United States of America; Universite de Geneve, SWITZERLAND

## Abstract

Grp94 and Hsp90 are the ER and cytoplasmic paralog members, respectively, of the hsp90 family of molecular chaperones. The structural and biochemical differences between Hsp90 and Grp94 that allow each paralog to efficiently chaperone its particular set of clients are poorly understood. The two paralogs exhibit a high degree of sequence similarity, yet also display significant differences in their quaternary conformations and ATPase activity. In order to identify the structural elements that distinguish Grp94 from Hsp90, we characterized the similarities and differences between the two proteins by testing the ability of Hsp90/Grp94 chimeras to functionally substitute for the wild-type chaperones *in vivo*. We show that the N-terminal domain or the combination of the second lobe of the Middle domain plus the C-terminal domain of Grp94 can functionally substitute for their yeast Hsp90 counterparts but that the equivalent Hsp90 domains cannot functionally replace their counterparts in Grp94. These results also identify the interface between the Middle and C-terminal domains as an important structural unit within the Hsp90 family.

## Introduction

The hsp90 family of molecular chaperones are essential for the survival of all eukaryotes. They are among the most abundant proteins in the cell, comprising over 2% of the total polypeptide mass, and are key responders to a variety of stress signals which include heat shock, glucose deprivation, ATP depletion, and oxidative stress [1–5]. In higher eukaryotes there are four hsp90 paralogs: cytosolic Hsp90α and Hsp90β, and two compartmentalized forms, Grp94, which is localized to the ER, and the mitochondrial paralog TRAP-1. More than 20 co-chaperones and over 500 clients for the cytosolic members have been identified to date [6–8]. Perhaps reflecting its more specialized role, the set of clients for Grp94 is far smaller and consists of proteins destined for secretion or surface expression, including the Toll-like receptors (TLRs) [4, 9], integrins [4], IgG [10, 11], insulin-like growth factors (IGF-I, IGF-II) [12], Wnt co-receptor LRP6[13] and cell surface TGFβ–anchoring molecule GARP [14].

**Fig 1:**
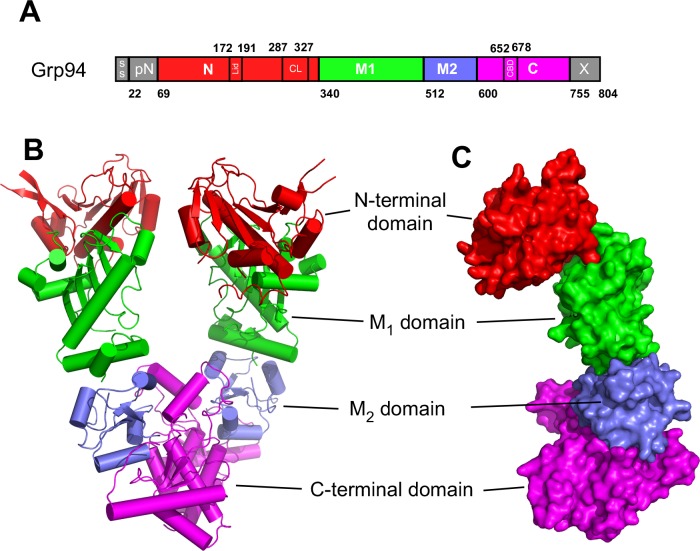
Domains and functional regions of hsp90 chaperones. The structure of Grp94 (PDB 2o1v) is shown. A) Schematic organization. B) Cartoon representation of the Grp94 dimer. C) surface representation of the Grp94 monomer.

All of the hsp90s exist as obligatory homodimers with each monomer consisting of three major structural domains: an N-terminal (N), a middle domain (M) and C-terminal (C) dimerization domain. Work over the past decade has shown that these can be further subdivided into 10 structurally or functionally distinct regions (**Fig 1**): 1) the signal sequence, found in Grp94 and Trap-1; 2) the pre-N domain, 3) the ATP binding N-terminal domain, 4) the helix 1,4,5 “Lid” that covers the ATP binding pocket of the N-terminal domain; 5) the charged linker at the end of the N-terminal domain; 6) M_1_, the first lobe of the Middle domain; 7) M_2_, the second lobe of the middle domain; 8) the C-terminal domain; 9) the second helix of the C-terminal domain, called the “client binding domain” (CBD) due to it’s role in binding some client proteins [15–17]; 10) the C-terminal extension (Cx) domain that contains a series of charged residues and the ER localization KDEL sequence (Grp94) or the TPR-binding MEEVD motif (Hsp90). In response to ATP binding and hydrolysis several of these elements undergo tertiary and quaternary structural rearrangements that are thought to be essential for the chaperone’s ability to bind and release clients and cochaperone partners (reviewed in [18]).

ATP binding and hydrolysis are essential for hsp90 function. However, despite having 47–50% sequence identity (73–81% similarity; percentages are given for the comparison between Grp94 and yeast Hsp90) and high overall similarity between their isolated N-, M-, and C-domain structures (RMSDs of 0.82–1.37 Å for Cα’s; Grp94:yHsp90), structural studies have elucidated different conformations for the nucleotide bound states of Hsp90 and Grp94 [19–26]. In response to ATP binding, Hsp90 adopts a fully closed double dimer conformation, where a second dimerization interface is formed via the two N-terminal domains and the “Lid” region is closed over the bound nucleotide. In contrast, ATP-bound Grp94 adopts a “twisted V” conformation, where the N-terminal domains are rotated about the N-M junction and sit diametrically opposed to one another, precluding the formation of the second dimer interface. In addition, instead of being closed, the Lid of nucleotide-bound Grp94 adopts an “extended open” conformation, swinging away from the bound ATP and exposing it to solvent. Assays of Hsp90 and Grp94 ATPase activity show that the two distinct Lid conformations correlate with a significant difference in the rates of ATP hydrolysis, with the cytosolic yeast paralog exhibiting a 5–25 fold higher rate of catalysis than Grp94 [20].

Given the high degree of sequence and structural similarity between the members of the hsp90 family, the observation of significant differences in the conformation and catalytic activity of the different paralogs is surprising. This prompted us to probe for the functionally important structural elements that distinguish the paralogs from each another. We have previously shown that the differences in the rates of ATP hydrolysis observed in full length Hsp90 and Grp94 could be ascribed to their N-terminal domains [20]. In this study, we characterize the similarities and differences between Hsp90 and Grp94 by testing the ability of Hsp90/Grp94 chimeras to functionally substitute for the wild type chaperones *in vivo*. From these studies, we show that some Grp94 domains can functionally substitute for their yeast Hsp90 counterparts but that the equivalent Hsp90 domains cannot replace their counterparts in Grp94. Furthermore, our data indicate that functional substitution does not correlate with the ATPase rate of the chimeric chaperone, and identifies the interface between the M and C domains as an important structural unit within hsp90s.

## Materials and Methods

### Cloning and chimera construction

All constructs used in these studies were derived from the yeast *HSP82* (Uniprot P02829) or canine Grp94 (Uniprot P41148) genes unless otherwise specified. Constructs were cloned into the NdeI/BamHI sites of pET15b for expression in bacteria or p414GPD that has been modified to attach an N-terminal His tag (GGHHHHHHGGH)[27] for expression in yeast (kind gift from D. Bolon, University of Massachusetts Medical Center). Grp94/Hsp90 chimeras were generated using cross-over PCR with primers that overlapped at the junction of the chimeras, and were confirmed by sequencing. The hspN-grpMC, grpN-hspMC and hspNM_1_-grpM_2_C chimeras were previously described [20].

### Protein production and purification

Proteins were expressed and purified as previously described [20]. Briefly, constructs were expressed in BL21Star (DE3) (Invitrogen) or Rosetta 2 (DE3) pLysS cells (Novagen) as N-terminal hexahistidine fusion proteins. The His-tags were retained unless specified. Cultures were typically grown at 37°C and induced at mid log with IPTG to a final concentration of 0.1–0.5 mM. The protein purification for all constructs consisted of Ni-affinity, Q-Sepharose anion exchange, and gel filtration purification steps. Purified protein fractions were concentrated to 30 mg/ml, aliqoted, and flash-frozen in liquid nitrogen. For ATPase assays samples were buffer exchanged using spin filters into 40 mM Hepes, pH 7.4, 150 mM KCl, and 5 mM MgCl_2_ and diluted to a final concentration of 50 μM before they were aliquoted, flash frozen in liquid N_2_ and stored at -80°C.

### Yeast complementation/viability and liquid growth assays

*S*. *cerevisiae* strain ECU82a is a haploid derivative of W303 in which both endogenous Hsp90 genes, *HSP82* and *HSC82*, have been knocked out (obtained from S. Lundquist, Whitehead Institute)[28]. The loss of both Hsp90 genes is a lethal genotype but ECU82a survives because it constitutively expresses wild type *HSC82* from pKAT6, a *URA3* marked high-copy plasmid. Chimeras and wild type Hsp90 were introduced as the sole source of hsp90 in yeast by plasmid shuffling. To test the ability of Grp94/Hsp90 chimeras to support growth, the genes encoding the chimeras were cloned into p414GPD, a *TRP* marked CEN plasmid with a strong constitutive promoter. Plasmids were introduced into ECU82a using the lithium acetate method and transformants containing chimeric constructs were selected on plates lacking tryptophan. Transformants were grown in liquid media lacking tryptophan to an OD_600_ of 0.6, serially diluted 5-fold and plated in the presence or absence of 5-FOA, which cures the cells of their original *URA3* vector. Plates were monitored for yeast growth at 22, 30 and 37°C for 3–9 days.

For liquid culture assays, strains were subjected to two rounds of selection on 5-FOA plates, then grown in SD-Trp media at 25°C and 30°C. Cultures were diluted to an OD_600_ of 0.1 upon reaching an OD_600_ of 0.8 to maintain log phase growth. A plot of the dilution-corrected OD_600_ versus time was fitted to an exponential equation to determine growth rates for each strain.

### Western blot analysis

The expression level of p414GPD-encoded Hsp90 constructs and chimeras in ECU82a was monitored by Western blot detection against the 6xHis tag at the N-terminal domains of the expressed proteins. Plasmids were transformed into ECU82a. Transformants were selected on SD -Trp -Ura plates and incubated at ≤30°C for 3 days. Colonies from fresh transformants were grown in SD -Trp -Ura media at 25°C overnight. Cells from 12–15 OD_600_ units of culture were collected by centrifugation and washed with ice-cold water. Cell pellets were flash frozen in liquid Nitrogen and stored at -80°C prior to lysis. Frozen cells were thawed on ice, resuspended in 50 mM Tris pH 7.6, 100 mM NaCl, 10 mM EDTA supplemented with 1 mM PMSF and Protease Inhibitor cocktail (Sigma P2714) and then lysed by vortexing with 0.5 mm glass beads at 4°C. SDS was added to a final concentration of 2% (v/v) and the lysates were immediately boiled for 5 minutes. After removal of cellular debris by centrifugation at 14000 rpm for 10 min at 4°C, the total protein concentration of each lysate was estimated using the bicinchoninic acid (BCA) protein assay. Lysates were stored in aliquots at -80°C prior to SDS-PAGE analysis and Western blots. For blotting, equal amounts of total protein (20 μg) were fractionated on a 4–15% SDS-PAGE gel. Following transfer to a PVDF membrane and blocking with instant milk, the expression of plasmid-derived chimeras was assessed by blotting against the 6xHis epitope tag using a mouse poly-His monoclonal antibody (Sigma H1029) followed by a mouse anti-IgG-HRP secondary antibody (Sigma A9044) and ECL detection (West Pico supersignal, Thermo PI34087). Blots were imaged using film luminography. Chimera 10 expression was detected using the HisG antibody (Fisher/Invitrogen R941-25) and the blot imaged with a ChemiDoc MP system (Biorad). After imaging, the PVDF membranes were stained with Coomassie blue to assess total protein loading.

### Ni-NTA pull-down assays with Hsp90 cochaperones

The interaction of mutant and chimeric Grp94/Hsp90 constructs with Hsp90 cochaperones was probed using a pull-down assay. Human Cdc37, yeast Aha-1 and human Hop were cloned into pET15b or pET28a and expressed as N- or C-terminally tagged hexahistidine fusion proteins. Protein production and purification protocols were similar to that described for the Hsp90/Grp94 constructs. Purified Hsp90, grpN-hspMC, hspNM_1_-grpM_2_C, hspNM_1_-grpM_2_C-hspCx, and Grp94 were digested with thrombin to remove their N-terminal His_6_ tags. Uncut material was removed by passage over a Ni-NTA column. Thrombin was removed from the cleaved chaperones using a p-Aminobenzamidine (Sigma) column and the cut material was further purified by gel filtration on an S200 column. Samples were collected and exchanged into binding buffer: 50 mM Tris pH 7.6, 100 mM NaCl and 1 mM β-Me. For the pull down assays, 200 μg of His_6_ tagged co-chaperone and 200 μg of untagged chaperone protein were mixed together, diluted to 200 μL with binding buffer, and incubated at 4°C for 1 h. 50 μL of this mixture was removed for the ‘Load’ sample aliquot. To the remainder, 30 μL (50% v/v slurry) of Ni-NTA resin that had been equilibrated in binding buffer was added and the mixture was nutated at 4°C overnight. Unbound material was removed by washing the resin three times with 20 resin volumes of 50 mM Tris pH 7.6, 100 mM NaCl, 20 mM Imidazole and 1 mM β-Me followed by a final wash step of 1 column volume. Samples were eluted with 30 μL of 50 mM Tris pH 7.6, 100 mM NaCl, 300 mM Imidazole and 1 mM β-Me. Prior to electrophoresis the eluent was diluted 5-fold with binding buffer to match the volume of the sample prior to the addition of the Ni-NTA resin. Protein samples were mixed with 1/5 volume of 6X SDS load buffer and equal volumes of each sample were resolved by SDS-PAGE on an 8–25% gradient Phast Gel (GE). Proteins were visualized by Coomassie blue staining.

### In vivo Grp94 analysis

#### Cell Lines

All Grp94 mutant-transduced Pre-B cell lines were generated from a parental Grp94-deficient E4.126 Pre-B cell line, which was a kind gift from B. Seed (Harvard). HEK293T-derived Phoenix Eco (PE) packaging cell line from ATCC was used for retrovirus production. All culture conditions were as previously described [29].

#### Antibodies and Peptides

The anti-FLAG antibody bioM2 used for the detection of FLAG-tagged Grp94 and chimeric variants was from Sigma. Biotin-conjugated anti-mouse CD11a (Clone: M174), CD49d (Clone: R1–2), TLR2 (Clone: 6C2), and TLR4 (Clone: MTS510) antibodies used for flow cytometry were purchased from eBioscience and were used to detect endogenous proteins.

#### Retrovirus Production and Transduction

C-terminally FLAG-tagged canine Grp94, Grp94 mutants, and Grp94/yeast Hsp90 chimeras were cloned into the NotI site of the bicistronic ecotropic retrovector MigR1, which also contains the enhanced Green Fluorescent Protein (EGFP) downstream of an IRES element. For retrovirus production, MigR1 constructs were transfected into the HEK293T-derived Phoenix Eco cell line using Lipofectamine 2000 (Invitrogen). Six hours after transfection, medium was replaced by pre-warmed fresh culture medium. Virus-containing medium was collected at 48 h after transfection. Harvested retrovirus was transduced into E4.126 Pre-B cells. To facilitate virus adhesion, spin transduction was performed at 1800 x g for 1.5 h at 32°C in the presence of 8 μg/ml hexadimethrine bromide (Sigma).

#### Flow Cytometry

All staining protocols, flow cytometry instrumentation as well as data analysis were performed as described without significant modifications [29, 30]. In brief, after retroviral transduction, cell surface expression of the Grp94 client (CD11a, CD49d, TLR2 or TLR4) was evaluated by staining with a client-specific biotin conjugated primary antibody at 4°C for 30 minutes. Detection was achieved by secondary antibody staining for 30 minutes at 4°C with a streptavidin-conjugated Allophycocyanin (APC) fluorescent antibody that binds to the biotin-conjugated primary antibody. A biotin-conjugated isotype antibody was used as a negative control to assess background levels. For intracellular staining, which was used to assess the overexpression level of FLAG-tagged Grp94 and the chimeras, cells were fixed in 4% paraformaldehyde at room temperature, and then permeabilized with ice-cold methanol at -20°C to allow antibody entry. Normal goat serum was used for blocking prior to anti-FLAG antibody or isotope control antibody staining. Antibody staining procedures were similar to surface staining, except all steps were done at room temperature. Antibody or isotype stained cells were acquired on a FACSCalibur (BD Biosciences) and analyzed using the FlowJo software (Tree Star). Using EGFP expression as a positive indicator for retroviral infection, the EGFP-positive cells were analyzed for both surface expression of matured Grp94 clients as well as for the intracellular expression of the Grp94 and grp/hsp chaperone chimeras.

### ATPase activity assays

ATPase activities were measured using two systems: (i) the PiPer phosphate Assay kit (Invitrogen) and (ii) an ATP-regenerating enzyme linked spectrophotometric assay system using lactate dehydrogenase (LDH) and pyruvate kinase (PK). Purified protein (Grp94, Hsp90 or chimeras) at a final concentration of 3–5 μM was added to the assay reagent mix plus varying concentrations of ATP (0–800 μM) and incubated for 90 or 120 minutes at 37°C. Fluorescence was measured at 544 nm/590 nm (excitation/emission) on a Molecular Devices SpectraMax Gemini XPS plate reader. Fluorescence readings were converted to free phosphate using a phosphate standard curve. Data was analyzed using the program Prizm and was fit to the Michaelis Menten equation to determine catalytic rates and K_M_.

The ATP regeneration coupled-enzymatic assay measures the hydrolysis of ATP as the production of ADP, which leads to the enzymatic conversion of NADH to NAD^+^. NADH but not NAD^+^ absorbs at 340 nm. The loss of the NADH signal is directly proportional to the ATP consumed. Enzymes and reagents were purchased from Sigma and the assay was performed as described previously [31–33].

## Results

### Some Grp94 domains functionally substitute for Hsp90 domains in yeast viability assays

The structures of the N, M, and C domains of Grp94 and yeast Hsp90 are remarkably conserved, with backbone R.M.S.Ds of 0.82 Å, 1.27 Å, and 1.37 Å for the individual N-N, M-M, and C-C domain comparisons, respectively. The amino acid sequences of the individual domains of the two chaperones are also highly conserved, with pairwise identities/similarities of 50.4/73.0% (N-N), 49.6/81.2% (M-M), and 48.3/74.8% (C-C). Given this conservation, we wanted to evaluate whether Grp94 domains could functionally complement their yeast Hsp90 counterparts. To this end, we made a series of Grp94/Hsp90 chimeras and tested whether these constructs could serve as the sole Hsp90 in yeast. Because yeast is dependent on Hsp90 for survival, viability was used as an indicator of functional complementation.

**Fig 2:**
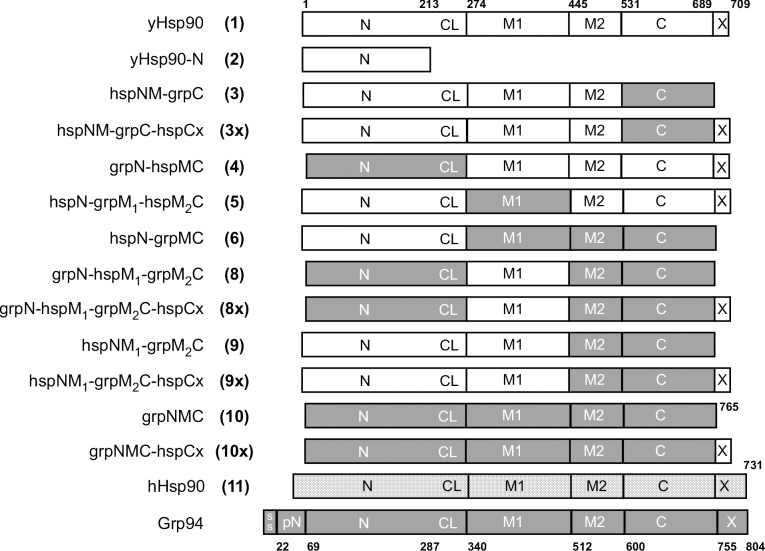
Chimeric constructs used in this study. Schematic representation showing the domain composition of Hsp90, Grp94, and the fragments used to create chimeras of Hsp90 and Grp94. Boundaries are shown with residue numbering for yeast Hsp90 and canine Grp94. The N-terminal chimeras referred to as grpN-hspMC (4), hspN-grpMC (3) and hspNM-grpM_2_C (9) were previously described in Ref. [20].

Chimeric Grp94/Hsp90 constructs were designed using domain boundaries derived from crystal structures of Grp94 and yeast Hsp90 and are shown schematically in **Fig 2**. All constructs contained N-terminal hexahistidine tags and were constitutively expressed from a Trp marked p414GPD plasmid that has been previously shown to accumulate Hsp90 to near wild-type levels [27, 34]. These plasmids were introduced into yeast strain ECU82a, which lacks both chromosomal copies of Hsp90 and instead expresses wild-type yeast Hsp90 from a 2 μm URA3 marked plasmid [28]. Upon selection with 5-FOA, the URA3 plasmid is expelled, leaving the Hsp90 or chimeric construct expressed from the p414GPD plasmid as the sole Hsp90 in the cell. p414GPD plasmids expressing wild type yeast Hsp90 (1–709) and human Hsp90α (1–732) served as positive growth controls, while a plasmid encoding the yeast Hsp90 N-domain (1–273) served as a negative growth control.

**Fig 3:**
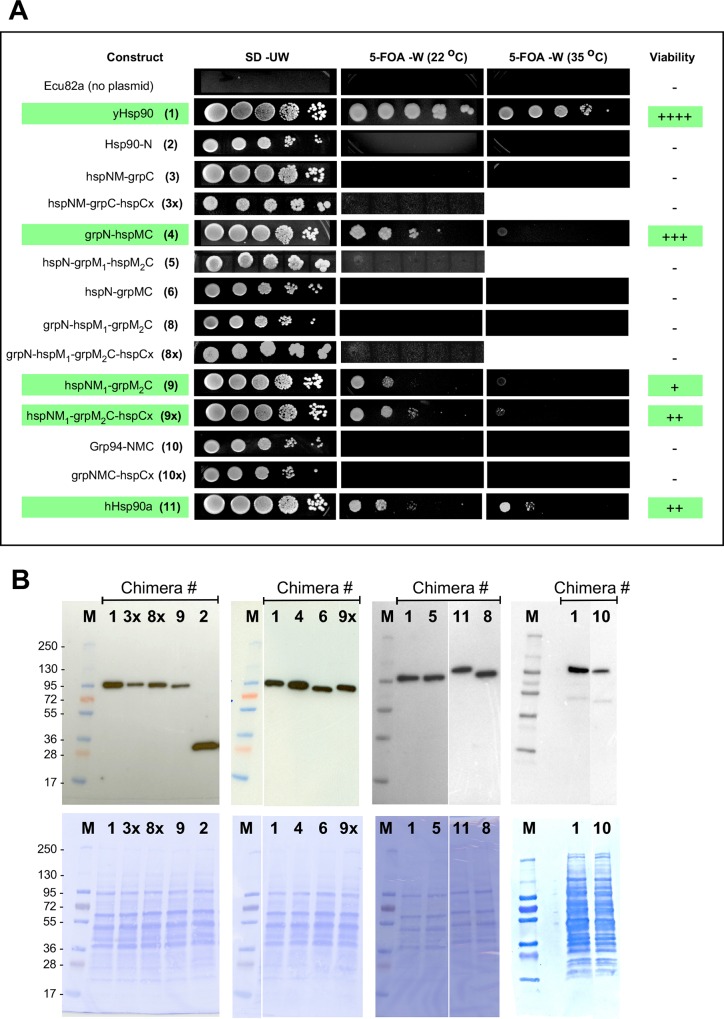
Select Grp94 domains support yeast viability. (A) Plasmid shuffle experiments demonstrate grpN-hspMC and hspNM_1_grpM_2_C are able to support yeast viability when expressed as the sole Hsp90. Cells were plated on FOA plates as 5-fold serial dilutions and were grown at the indicated temperatures for 3–9 days. Images are from day 4. Green highlights indicate positive complementation. (B) Western blot of yeast whole cell lysates. 20 μg of total protein extracted from the lysis of plasmid-transformed yeast cells grown in SD–Ura -Trp was separated by SDS-PAGE. The expression level of plasmid-expressed hsp90 chimeras was evaluated by Western blotting against the N-terminal His_6_ epitope tag. Lysate from Chimera 1 (yeast Hsp90) was included on each blot as a transfer and antibody control. Coomassie blue stained PVDF membranes used for blotting are displayed below their respective luminographs to allow comparison of total protein levels.

Under 5-FOA selection, where the p414GPD-encoded gene product is the sole Hsp90, some, but not all, Grp94 domains can functionally substitute for their yeast Hsp90 counterparts (**Fig 3A).** Of the eleven Grp94/Hsp90 chimeras tested, only the following three supported yeast growth: the grpN-hspMC (4), the hspNM_1_-grpM_2_C (9), and the hspNM_1_-grpM_2_C-hspCx (9x) chimeras. Importantly, although the growth rate of some of these Grp94/Hsp90 chimeras was slower than that of wild type yeast Hsp90, these chimeras still represent functional substitution. This is because the chimeras that failed to support yeast viability produced no evidence of yeast growth even after two weeks of incubation and thus constitute true negatives. The Grp94/Hsp90 chimeras that failed to support yeast viability include the hspNM-grpC (3) chimera that contains both the complete yeast Hsp90 ATP binding site and several co-chaperone binding sites, the hspN-grpMC (6), the near full-length Grp94-NMC (10) constructs, and any chimera containing the Grp94 M_1_ domain (5,8).

The addition of the hspCx region onto the hspNM_1_-grpM_2_C chimera (9) (hspNM_1_-grpM_2_C –hspCx) showed a modest but reproducible improvement in the growth rate of this chimera compared to the same chimera lacking the hspCx region (9x vs 9). The hspCx region contains the MEEVD sequence recognized by TPR domain containing co-chaperones and has been implicated in aiding the anti-aggregation properties of Hsp90 chaperones, thereby promoting robust cell growth [35, 36]. We thus investigated whether addition of the hspCx region could impart viability onto chimeras that were previously unable to support yeast survival. As seen in **Fig 3A**, the addition of the hspCx tail onto Grp94-NMC (10→10x) or the hspNM-grpC (3→3x), or the grpN-hspM_1_-grpM_2_C (8→8x) chimeras failed to restore the viability of these constructs.

In case the results from the yeast viability assays are due to wide differences in chimeric protein expression levels, we used Western blots of cell lysates to confirm that the expression levels of the His_6_ tagged Grp94/Hsp90 chimeras are similar to that of wild-type yeast Hsp90 (1) expressed from the p414GPD plasmid (**Fig 3B**). To rule out protein instability as the source of complementation failure, we also purified a subset of both complementing and non-complementing Grp94/Hsp90 chimeras and tested these for ATP hydrolysis activity (**Table 1**). In all cases ATPase activity was observed. In agreement with previous observations [20], the ATPase rate was dictated by the identity of the N-terminal domain in the chimera. Interestingly, the ability of a Grp94/Hsp90 chimera to support yeast viability is not related to the rate of ATP hydrolysis. As seen in **Table 1**, chimeras with low rates of ATPase activity (e.g. grpN-hspMC (4)) support yeast viability, while chimeras with wild type ATPase activity (hspN-grpMC (6) or hspNM_1_-grpM_2_C (9)) either fail to support yeast viability (chimera 3) or produce very slow rates of growth (chimera 9). This lack of correlation is not restricted to chimeric chaperones, since the ATPase rate of human Hsp90α is also considerably lower than that of yeast Hsp90 [37], and this homolog supports yeast viability. Taken together, the inability of a tested chimera to support yeast growth is therefore suggestive of functional differences between Grp94 and Hsp90 in the substituted domains.

**Table 1:** Relative ATPase rates of Grp94/Hsp90 chimeras.

Protein	Relative ATP hydrolysis rate
Yeast Hsp90 (1)	100
grpN-hspMC (4)	20
hspN-grpMC (6)	95
hspNM_1_-grpM_2_C (9)	90
Grp94-NMC-hspCx (10x)	20
Grp94-NMC (10)	20

Although the grpN-hspMC (4) and hspNM_1_-grpM_2_C (9) chimeras functionally substitute for yeast Hsp90, the apparent growth of yeast dependent on these chimeras as the sole Hsp90 was slower than that of cells expressing the wild-type chaperone. As seen in **Fig 3A**, at 22°C, cells expressing the wild-type yeast Hsp90 grow fastest, followed closely by grpN-hspMC (4), and then the hspNM_1_-grpM_2_C-hspCx (9x), hspNM_1_-grpM_2_C (9) and human Hsp90α constructs. It was surprising that the grpN-hspM_1_-grpM_2_C chimera (8) failed to achieve viability, since it contains the two Grp94 domains that individually could substitute for their yeast Hsp90 counterparts (grpN, grpM_2_C), albeit with a slower growth profile. It is likely that the additive effect of two growth-retarding substitutions is the reason why the grpN-hspM_1_-grpM_2_C (8) chimera, did not result in viable yeast. The addition of the hspCx tail, which we found to result in a slightly better growth with chimera 9, also failed to impart yeast viability to chimera 8 (8 vs 8x).

Interestingly, the growth rate of yeast harboring the Grp94/Hsp90 chimeras that did yield viable organisms equals or exceeds the rate exhibited by yeast expressing human Hsp90α (11) as the sole Hsp90 (**Fig 3A**), suggesting that the Grp94/Hsp90 chimeras are behaving as authentic chaperones. Similar relative growth rates were observed at 30°C. Significant differences in the survival of yeast harboring Grp94/Hsp90 chimeras were only seen at 35°C and above, suggesting a divergence in the stress response between the chimeras and the wild type yeast Hsp90.

**Fig 4:**
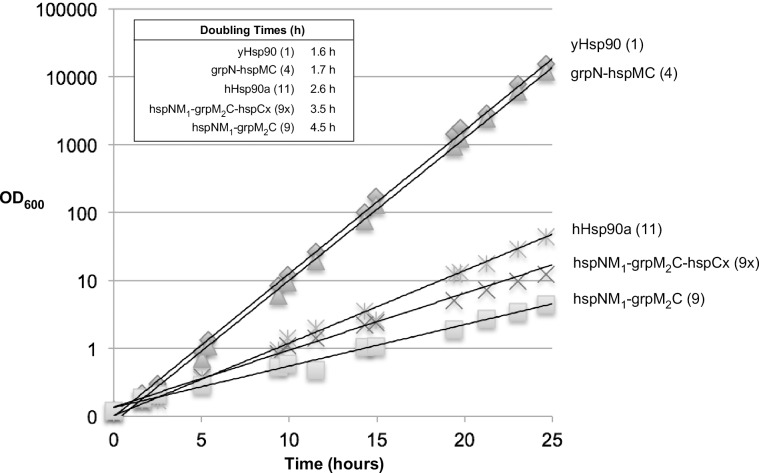
Growth in liquid media of ECU82a expressing yeast Hsp90, grpN-hspMC, human Hsp90a, hspNM_1_grpM_2_C, and hspNM_1_grpM_2_C-hspCx as its sole Hsp90. Cultures were started at a cell density of 3.5 x 10^6^ cells per mL in YPD medium (time 0) and incubated with shaking at 30°C. Data represents an average OD_600_ from two yeast clones.

To further evaluate the growth differences between wild-type and chimeric hsp90s, we grew the chimera-containing strains that supported yeast viability in liquid culture and monitored their doubling times. As seen in **Fig 4**, the liquid culture growth curves show two classes of growth. Yeast harboring wild-type yeast Hsp90 and the grpN-hspMC (4) chimera grew at nearly identical rates, with doubling times of 1.6 and 1.7 hours, respectively. Yeast harboring the hspNM_1_-grpM_2_C-hspCx (9x) and hspNM_1_-grpM_2_C (9) chimeras or human Hsp90α (11) exhibited slower growth, with doubling times of 3.5, 4.6, and 2.8 hours, respectively. These results agree qualitatively with the growth rates observed on solid media, although the differences in the relative growth rates between chimeras are reduced when grown in liquid media. This phenomenon has been observed previously for heterologous Hsp90’s expressed in yeast [38], and may reflect different chaperone substrate requirements for different environmental conditions.

Taken together, these experiments show that the Grp94-N and Grp94-M_2_C domains can each functionally substitute for their yeast Hsp90 counterparts, but the intact Grp94, the Grp94 M_1_ domain, and the Grp94-C domain cannot.

### Grp94/Hsp90 chimeras interact with Hsp90 cochaperones

Hsp90 depends not only on nucleotide induced conformational changes for client activation but also relies on cochaperones to facilitate different stages of the chaperoning cycle. Hsp90 has at least eighteen known cochaperones [39], but in Grp94 only two—Cnpy-3 and MZB1—have been identified to date [40, 41]. The Grp94 cochaperones bear no similarity to any of the Hsp90 cochaperones. Although the individual domains of Grp94 and Hsp90 exhibit a high degree of structural and sequence homology, chaperone-cochaperone interactions are likely to be sensitive to small differences in sequence and structure. Given that the Grp94/Hsp90 chimeras we identified as viable were comprised of juxtaposed domains from different paralogs, we were interested in determining how much co-chaperone binding and discrimination was retained by these constructs.

**Fig 5:**
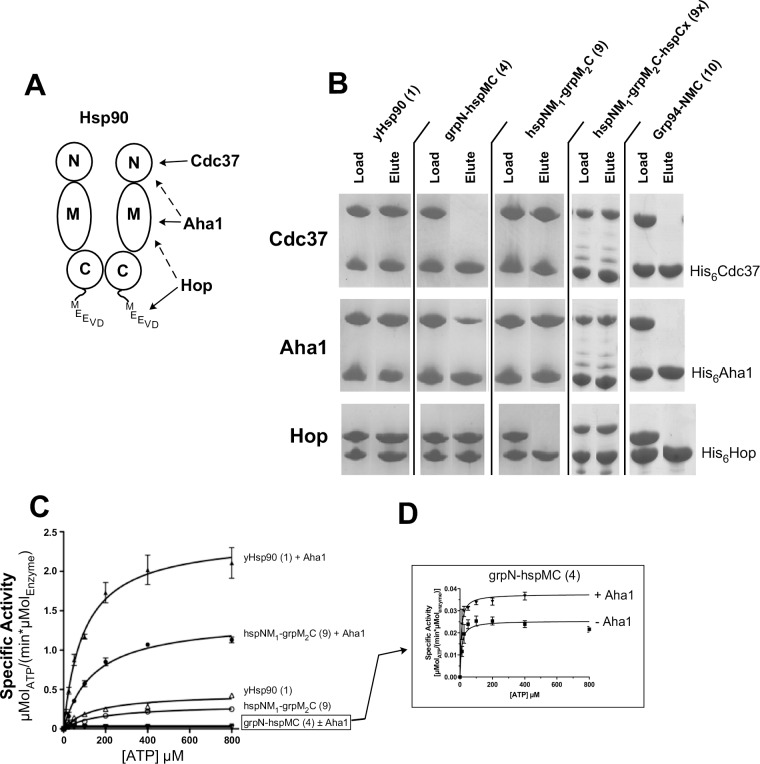
Grp94/Hsp90 chimeras interact with Hsp90 co-chaperones. (A) Cartoon summary of Hsp90:cochaperone binding loci. (B) Ni-NTA pull-down assay shows that His_6_-Cdc37 interacts with hspNM_1_-grpM_2_C and Hsp90 but not grpN-hspMC; His_6_-Hop interacts with grpN-hspMC and Hsp90 but not hspNM_1_-grpM_2_C; His_6_-Aha1 interacts with hspNM_1_-grpM_2_C, Hsp90, and partially with grpN-hspMC. Equal amounts of untagged chaperone and His-tagged cochaperone were mixed together and incubated for 1 h at 4°C followed by overnight incubation with Ni-NTA resin. The Load sample was removed prior to the addition of Ni-NTA resin. Unbound protein was removed by sequential washes of buffer containing 20 mM imidazole. Bound proteins and protein complexes were eluted with buffer containing 300 mM imidazole and resolved in parallel with the Load sample on SDS-PAGE gels. (C) hspNM_1_-grpM_2_C ATPase activity is stimulated 5-fold by Aha1. Wild-type Hsp90 is stimulated 10-fold whereas grp-hspMC is stimulated a modest 1.5-fold (D). ATPase activity was monitored by a NADH coupled enzymatic assay system, which measures the consumption of NADH as a function of ADP released by Hsp90s. Assays were measured with or without the addition of cochaperone Aha1 at a 1:2 molar ratio of hsp90:Aha1. Wild-type Aha1 alone did not exhibit ATPase activity (not shown).

His-tagged yeast cochaperones Cdc37, Aha1, and Hop were tested for their ability to bind untagged Grp94, the grpN-hspMC chimera (4), and the hspNM_1_-grpM_2_C chimera (9) using a Ni-NTA pull down assay. Previous studies of purified components have shown that Cdc37 interacts with the N-terminal domain of Hsp90 [42], Aha1 interacts strongly with the middle domain and less strongly to the N-domain [43–46], and Hop interacts with both the C-terminal MEEVD TPR binding motif and the middle domain [47–51] (**Fig 5A**). Thus, these three cochaperones are useful in determining whether the substituted domains in these positive chimeras retain the architecture for cochaperone binding similar to that of wild type yeast Hsp90. As seen in **Fig 5B**, none of the yeast cochaperones interacts with full length Grp94, as expected, indicating that the Grp94 domains do not possess the required recognition surfaces for cochaperone binding. Chimeras containing the yeast Hsp90 domains that are targeted by the cognate cochaperones did bind to their cochaperone partners, confirming the overall structural integrity of the chimeric constructs. Thus, grpN-hspMC (4) interacts with Aha1 and Hop, and hspNM_1_-grpM_2_C (9) binds to Cdc37 and Aha1 (**Fig 5B)**. When the hspCx was added to hspNM_1_-grpM_2_C (9), this chimera (9x) now also interacts with Hop.

Aha1 has been shown to interact with both lobes of the Middle domain of Hsp90 as well as with the N-terminal domain. From **Fig 5B**, we note that while the hspNM_1_-grpM_2_C chimera (9), which contains the non-cognate Grp94 M_2_ lobe, exhibits robust pulldown with Aha1, the pulldown of the grpN-hspMC chimera (4) with Aha1 was considerably weaker. Since Aha1 is a potent stimulator of the ATPase activity of Hsp90, we tested whether the inclusion of non-cognate Grp94 domains into the two chimeras would affect Aha1’s stimulation of the ATPase activity of the chaperone chimeras. As seen in **Fig 5C**, both of the chimeras that contain Grp94 domains exhibit weaker stimulation of their ATPase activity compared to that of Hsp90. While Aha1 stimulates the ATPase activity of wild type yeast Hsp90 by 10-fold, the hspNM_1_-grpM_2_C chimera (9), which contains the non-cognate Grp94 M_2_ domain, exhibits only a 5-fold stimulation by Aha1. When the grpN-hspMC chimera (4) was tested, only a 2-fold stimulation of the ATPase activity by Aha1 was observed (**Fig 5D**).

Together these data indicate that the tested Hsp90 cochaperone proteins are specific partners for their Hsp90 domains. Grp94 domains incorporated into Grp94/Hsp90 chimeras are not compatible substitutes. Nevertheless, despite the juxtaposition of Grp94 domains into these chimeras, the Grp94/Hsp90 chimeras maintain the overall structural features that support the details necessary for the recognition of their cognate cochaperones.

### Hsp90 domains do not functionally substitute for Grp94 domains

In the yeast studies reported above, two of the Grp94/Hsp90 chimeras we tested were capable of supporting yeast viability, indicating that Hsp90 domains can be replaced with their Grp94 counterparts and still maintain their structural integrity and function. We thus asked whether the reciprocal substitution of the equivalent yeast Hsp90 domains–the N and M_2_C domains—into Grp94 allowed for retention of Grp94 function *in vivo*.

Yeast and other lower eukaryotes do not have a Grp94 homolog. Consequently, in order to test the ability of Grp94/Hsp90 chimeras to functionally substitute for Grp94, we used flow cytometry to monitor the maturation and cell surface expression of Toll-like receptor (TLR2, TLR4) or integrin (CD11a, CD49d) client proteins in a Grp94-deficient pre-B cell line that was transduced with a Grp94 or chimera-containing retrovirus. Because TLRs and integrins are strictly dependent on Grp94 for maturation [4, 9], the rescue of client protein maturation after transient expression of Grp94 or a Grp94/Hsp90 chimera indicates functional substitution.

To carry out the assay, Grp94 or the chimeric constructs were cloned into a retroviral vector that also contained the coding sequence for the fluorescent marker protein EGFP. Transfection into Phoenix Eco packaging cells yielded murine stem cell retrovirus (MCSV) containing the bicistronic chaperone-EGFP insert. The excreted retrovirus was recovered from the culture medium and used to infect a Grp94-deficient pre-B cell line. This cell line fails to express Grp94 clients unless a functional Grp94 is supplied by the infecting retrovirus. After allowing for viral replication and chaperone expression, the transduced cell lines were stained for cell surface expression of matured client protein or the intracellular expression of chaperone using fluorescently conjugated antibodies and analyzed by flow cytometry. EGFP fluorescence was used as a marker for retroviral infection. EGFP positive cells were gated, assayed for the fluorescent signal from the antibody staining, and the results plotted as normalized cell count vs. fluorescence histograms. Positive signal was judged by comparison with cells stained with an isotype control antibody.

**Fig 6:**
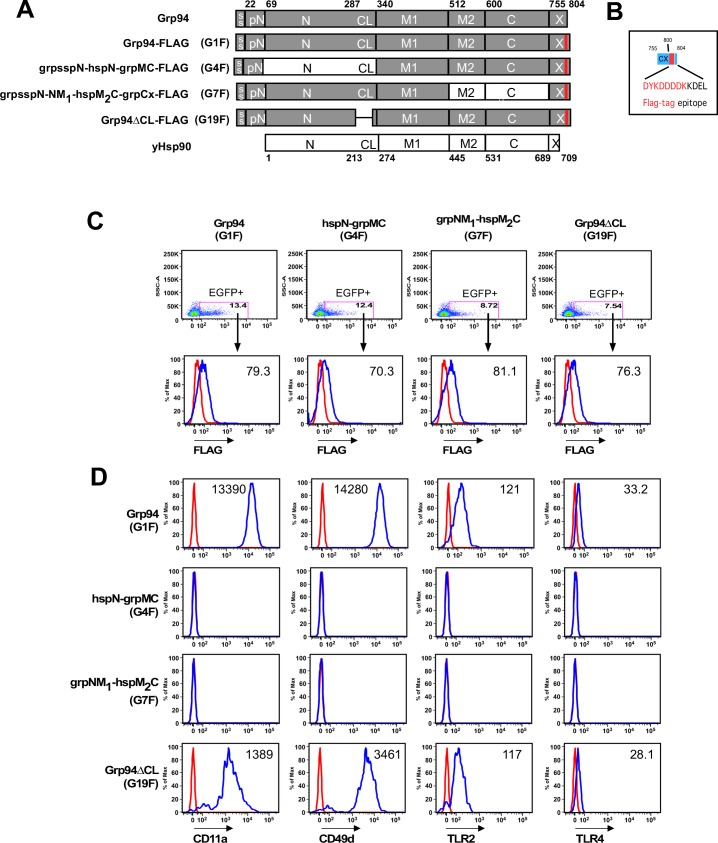
GRP94 client expression with Grp94/Hsp90 chimeras. A) Schematic representation of constructs tested. All constructs contained the Grp94 signal sequence (ss), the preN-domain, and the KDEL ER retention sequence found in the Grp94 C-terminal extension (Cx). The FLAG tag is shown as a red bar. B) Detail of the FLAG tag incorporated into each tested construct. C) EGFP gating and intracellular staining of Grp94-null E4.126 cells virally transduced with Grp94 constructs. Top panel: dot plot of the side scattering intensity (SSC-A, Y-axis) of analyzed cells (a measure of cell size and clustering) vs. the fluorescence of the co-expressed EGFP (X-axis). The number of events is represented by the color of the dots. The dots in the magenta box correspond to EGFP positive cells and the numbers show the percentage of all cells that were assigned to this gate. Lower panel: Histogram of the normalized cell count vs. conjugated anti-FLAG (blue) or isotype control (red) fluorescence. Fluorescence intensity is proportional to the copy number of the antibody target. Numbers represent the mean fluorescence intensity of the blue histograms. D) Cell surface expression staining of Grp94 clients as a function of virally transduced Grp94 construct. Cells were gated for EGFP expression as in C). Red = isotype control antibody histogram, blue = anti-client antibody histogram. The mean fluorescence intensity of the blue histogram is indicated.

We designed the reciprocal equivalents of the grpN-hspMC (4) and hspNM_1_-grpM_2_C (9) chimeras that restored yeast viability, producing the hspN-grpMC (G4F) and grpNM_1_-hspM_2_C (G7F) chimeras (**Fig 6A**). To allow for the quantitation of chaperone expression with a common antibody, all constructs contained a FLAG tag epitope inserted immediately upstream of the terminal KDEL sequence (**Fig 6B**). A FLAG tag in this position does not interfere with Grp94 function [29].

As seen in **Fig 6D**, the hspN-grpMC chimera (G4F) fails to support integrin or TLR maturation since the histograms of client and isotype antibody staining are fully overlapped. In an earlier report by Randow and Seed [4], a Grp94 chimera containing the human Hsp90 N domain was unable to substitute for Grp94 in the maturation of TLRs or integrins [9]. Because the C-terminal boundary of the Grp94 N-terminal domain was incorrectly assigned to end within strand 8 of Grp94 (residue 282) rather than after its paired beta strand, strand 9 (residue 337) when this analysis was originally reported, we considered the possibility that the failure of the shortened Hsp90 N domain (hspN’ (residues 1–208)) to substitute for the Grp94 equivalent in an hspN’-grpMC chimera was due to the non-cognate pairing of hHsp90 and Grp94 beta strands in the original chimeric construct. To address this issue, the hspN-grpMC (G4F) chimera we designed contained the complete yeast Hsp90 N-domain (residues 1–273), including the charged linker, using the available crystal structure models to accurately assign the domain boundaries. Despite the inclusion of authentic domain boundaries, however, the hspN-grpMC chimera (G4F) still fails to support integrin or TLR maturation. As a control, and in agreement with the earlier report, both full length Grp94 (G1F) and Grp94ΔCL (G19F), a Grp94 deletion construct lacking only the charged linker domain, show client maturation. To control for false negatives arising from the differences in the expression of the virally transduced Grp94 constructs, we measured the level of each Grp94 or Grp94/Hsp90 chimera by flow cytometry using intracellular staining against the common FLAG-tag epitope (**Fig 6C**). As judged by the mean fluorescent intensity of the observed histograms, all Grp94 constructs were expressed at equivalent levels. Together these data suggest that the Hsp90 N-domain cannot substitute for its Grp94 counterpart.

Similarly, when the yeast Hsp90 M_2_C domain replaced the equivalent region in Grp94, the resulting chimera–grpNM_1_-hspM_2_C-grpCx (G7F) could not support integrin or TLR maturation either (**Fig 6**). The addition of the Grp94 CBD element [17] to this chimera did not restore client protein maturation (data not shown). Together with the failure of Hsp90 N domain substitutions to function in Grp94 chimeras, these results suggest that Grp94 clients have a stringent requirement for Grp94 domains for Grp94 function.

## Discussion

Previous genetic complementation studies have shown that yeast viability in the absence of the yeast wild type chaperone can be conferred by heterologous cytosolic Hsp90s including those from human, drosophila, trypanosomes, *C*. *elegans*, *Candida*, and *P*. *falcifurum* [38, 52–56]. Here, we now report that chimeric Hsp90s with selected domain substitutions from the ER paralog Grp94 also support yeast viability. Our results show that yeast Hsp90 chimeras containing the Grp94-N or Grp94-M_2_C domains can function as the sole Hsp90 in yeast. To our knowledge, this is the first demonstration that domains of Hsp90 paralogs have the potential to functionally complement one another.

Under non-stress conditions the growth rate of yeast harboring the grpN-hspMC chimera equaled that of yeast with wild type Hsp90. Yeast bearing the hspNM_1_-grpM_2_C chimera construct or human Hsp90α were also able to support viability, but both exhibited slower growth rates and poor stress response. In noted contrast to the domain swaps assayed in yeast, no equivalent reciprocal chimeras, where complete yeast Hsp90 domains replaced their Grp94 counterparts, were able to support the maturation of Grp94 client proteins.

### N-domain substitutions in yeast Hsp90

It was surprising that the grpN-hspMC chimera (4) could function as the sole heterologous hsp90 in yeast. Biochemically, replacing the yeast N-domain with Grp94-N results in chimeric chaperones with only 20% of the ATPase rate of wild type yeast Hsp90 [20]. Structurally, the N-domains of yeast Hsp90 and Grp94 exhibit the greatest conformational differences among all the domains of the two chaperones [23]. The fact that these differences do not affect the viability of yeast harboring the grpN-hspMC chimera (4) suggests that the observed structural and biochemical differences in the Grp94 and Hsp90 N-domains are functionally irrelevant to yeast, and that the rate of ATP hydrolysis is also of minor importance in determining viability.

### M-domain substitutions in yeast Hsp90

**Fig 7:**
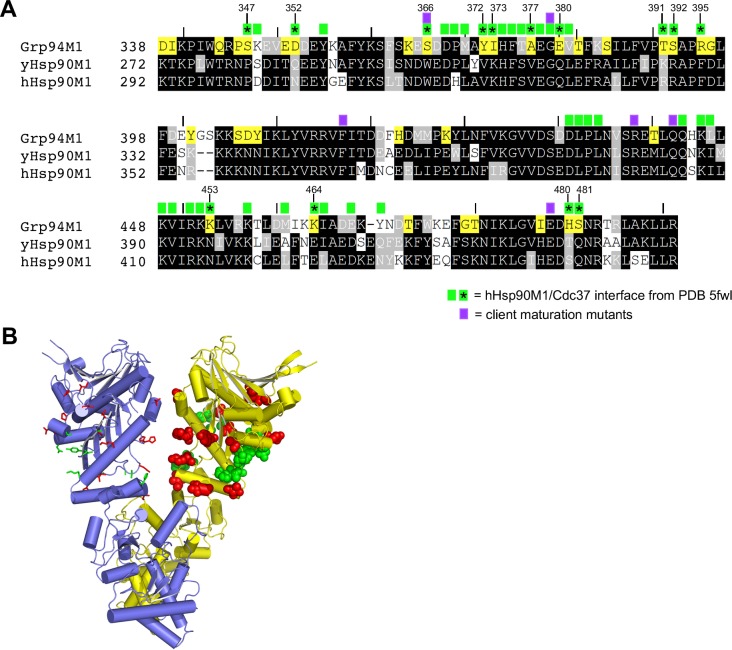
The M_1_ domain of Grp94 and Hsp90 differ in their surface residues. A) Alignment of the M_1_ lobe of the M domains of Grp94, yeast Hsp90, and human Hsp90. Grp94 residues highlighted in yellow differ from the yeast and human Hsp90 consensus. Purple boxes indicate residues shown to be mutagenically sensitive in yeast Hsp90 for client maturation. Green boxes indicate residues of human Hsp90 that interact with Cdc37 in the cryo-EM structure of Cdk4-Cdc37-Hsp90 (PDB code 5fwl). Asterisks and numbers (Grp94 numbering) highlight Cdc37-interacting residues that differ between Hsp90 and Grp94. B) Mapping the unique residues onto the structure of the Grp94 dimer. PDB code 2o1v was used. Residues highlighted in yellow in A) are shown as sticks or spheres on the on protomers A and B, respectively. Green colored residues correspond to residues highlighted with asterisks in panel A.

Our results show that the M_1_ lobe of the Grp94 M-domain could not substitute for the M_1_ lobe of yeast Hsp90 in any chimeric construct. Structurally, the M_1_ domains of Grp94 and yeast Hsp90 show good alignment, with an RMSD of 0.99 Å for backbone Cα’s, suggesting that the conformation of the lobe is not the reason for its failure to complement. To further probe the origins of this phenomenon, we aligned the sequences of the M_1_ domains of human Hsp90 and yeast Hsp90 with the M_1_ domain of Grp94 (**Fig 7A**). Because human Hsp90 complements yeast Hsp90, we reasoned that identifying residues of Grp94 that did not match the consensus between human and yeast Hsp90 might reveal the basis for the failure of the M_1_ domain of Grp94 to complement. As seen in **Fig 7A**, 34 out of 173 M_1_ domain residues differ between Grp94 and the yeast/human Hsp90 consensus. Mapping these 34 residues onto the structure of Grp94, it is apparent that they are evenly distributed across the M_1_ domain and, with one exception (Ser385), are all exposed on its surface. These residues thus have the potential to interact with client proteins or cochaperones.

The M_1_ domain is a locus for both client and cochaperone interactions, which may explain its sensitivity to substitution. Both lobes of the M-domain as well as the second helix of the C-domain have been implicated as sites of client binding [57, 58]. Previous studies have also identified six point mutants in the yeast Hsp90 M_1_ domain that are important for v-src or glucocorticoid receptor maturation. These include Trp300, Gly313, Phe349, Arg380, Gln384, and Glu431 [28, 59, 60]. Interestingly, within this set, five of the residues are conserved among paralogs, but Trp300, which is essential for proper v-src maturation [60], is replaced by Ser366 in Grp94 and may represent a deleterious substitution. Finally, a recent cryo-electron microscopy study of a complex between protein kinase Cdk4, the cochaperone Cdc37, and human Hsp90 showed that the beta 5 strand of the kinase client is clamped between the two opposed middle domains of the Hsp90 dimer in the client-chaperone complex. The Cdc37 in this complex is also wrapped around the M_1_ domain [61]. Together these data point to the essential role of the M_1_ domain in client protein maturation. The observation that the Grp94 M_1_ domain cannot substitute for that of yeast Hsp90 is thus consistent with the notion that client-specific interactions depend on the precise surface characteristics of the M_1_ domain rather than on its conserved overall fold.

### Cochaperone interactions

The pulldown data presented here show that the Hsp90 cochaperones Cdc37, Aha1, and Hop/Sti1 do not interact with the Hsp90 chimeras containing Grp94 substitutions of the domains they target. This failure does not reflect a loss of the overall structural integrity of the chimeric chaperones, since chimeras with substitutions of Grp94 domains outside of the cochaperone binding/recognition region retain cochaperone binding. Instead, the data indicate that, despite the conformational similarity of individual Grp94 and Hsp90 domains, precise sequence specific interactions govern the binding of cochaperones to their cognate Hsp90 domains.

Unlike many of the other Hsp90 cochaperones such as Aha1 and Hop (Sti1), Cdc37 is strictly required for yeast viability [62, 63]. Surprisingly, however, the grpN-hspMC chimera (4), which does not bind Cdc37 in our pulldown assay, nonetheless supports yeast viability. The Hsp90 binding site for Cdc37 was originally mapped to the N-terminal domain [42], but recent data from the cryo-EM structure of the Cdk4-Cdc37-hHsp90 complex [61] reveals that, in the context of the client assembly, Cdc37 makes the vast majority of its interactions with the M_1_ domain of the chaperone (**Fig 7A**). Thus, although the grpN-hspMC chimera (4) lacks the cognate Hsp90 N-terminal domain, it does contain the alternate Hsp90 M_1_ binding site for Cdc37 that is used in the context of client complexes. This may explain the ability of the grpN-hspMC chimera (4) to functionality substitute for Hsp90 despite lacking the N-domain Cdc37 binding site. Similarly, a comparison of the M_1_ domains from Grp94 and Hsp90 shows that 14 of the 41 M_1_ domain residues that interact with Cdc37 are not conserved between Hsp90 and Grp94 (**Fig 7A**). These non-conserved residues are distributed evenly over the surface of the Grp94 M_1_ domain (**Fig 7B**). This divergence between the M_1_ domains of Grp94 and Hsp90 may preclude Cdc37 binding to the Grp94 M_1_ domain in the client bound complex, and, if so, helps reconcile the observation reported here that the yeast Hsp90 M_1_ domain is required for viability in all tested grp/hsp chimeras.

Although the pulldown data show that Aha1 binds to the grpN-hspMC (4) and hspNM_1_-grpM_2_C (9) chimeras, the ATP hydrolysis results show that Aha1 fails to stimulate ATP hydolysis in these chimeras to the degree that it stimulates ATP hydrolysis in wild-type yeast Hsp90. This may indicate a weakened functional interaction between Aha1 and these chimeras. Interestingly, Aha1 is not essential for viability at mesophilic temperatures but is required for yeast viability under conditions of organismal stress, including heat shock and inhibitor treatment [45]. The weakened interaction between Aha1 and the grpN-hspMC (4) and hspNM_1_-grpM_2_C (9) chimeras may be a contributing factor in the failure of these chimeras to support yeast viability at elevated temperatures.

### C-domain substitutions in yeast Hsp90

Like the M_1_ domain, the C-terminal domains of yeast Hsp90 and Grp94 are also structurally conserved, with backbone R.M.S. deviations of less than 1 Å. The fact that the yeast complementation data showed that the C-domain of Grp94, with or without the appended hspCx tail, did not substitute for its yeast Hsp90 counterpart at first appeared to parallel the complementation results obtained for the M_1_ domain. However, while the hspNM-grpC and hspNM-grpC-hspCx chimeras both failed to support yeast viability, the hspNM_1_-grpM_2_C chimera did support yeast growth, indicating that the presence of the Grp94-C domain was not the source of the hspNM-grpC failure. To explain this apparent contradiction, we examined the structure of the C-terminal domain in the context of the intact chaperone. The boundary for the C-domain in Hsp90 was initially established by limited protease digestion studies, and the stability of the isolated C-domain was consistent with its assignment as an independent structural entity [16, 60, 64]. The complementation data presented here, however, suggests that in the context of the larger chaperone, the functional C-terminal unit is the entire M_2_C segment. In support of this, an examination of existing crystal structure data from all hsp90 family members indicates that the M_2_ and C domains form a tightly integrated structural unit. The M_2_-C interface is extensive, burying on average 2284 Å^2^ of surface per protomer, compared to the M_1_-M_2_ interface, which buries only 1325 Å^2^ per protomer (**Table 2**). The underlying protein backbone similarity of the yeast Hsp90 and Grp94 domains allowed us to construct a model of the likely yeast hspM_2_-grpC interface. Inspection of this chimeric model reveals potential steric clashes between the side chains of the yeast Hsp90-M_2_ domain (Thr525, Leu530, Glu531, Glu532, Thr533) and the juxtaposed Grp94-C domain (Lys603, Thr604, Lys605, Arg639). The disruption of the authentic, structurally conserved M_2_C interface in the hspNM-grpC chimera may explain the failure of this construct to function as an hsp90 in yeast. This analysis is supported by a recent molecular dynamics simulation study [65] that also concluded that the M_2_C is the functional structural unit at the C-terminal end of the hsp90 paralogs.

**Table 2:** Solvent Accessible Surface Area of Domain Interfaces.

Protein	M_2_/C	M_1_/M_2_	N/M_1_
Grp94 (PDB 2o1v)	2591	1209	1284^1^
yHsp90 (PDB 2cg9)	2053	1414	1982
HtpG (PDB 2ioq)	2208	1347	1459
Trap-1 (PDB 4ipe)	1098^2^	1330	2118
Average	2284	1325	1389

^1,2^ portions of the protein near these interfaces were disordered and were not included in the calculation of average area

### Domain substitution and Grp94 function

In contrast to the result showing that the Grp94-N and Grp94-M_2_C domains can functionally substitute for their yeast Hsp90 counterparts, none of the corresponding yeast Hsp90 domains were found to functionally substitute for their Grp94 counterparts in tests of Grp94 client maturation. In this regard, the failure of the major yeast Hsp90 domains to complement their Grp94 counterparts resembles the effect of Grp94 domain substitutions on the ability of yeast Hsp90 to interact with its cochaperone proteins. The yeast cochaperones form highly specific interactions with Hsp90 and cannot tolerate the substitution of the Grp94 equivalent of the domains they target. By analogy to this example, it is possible that the interaction of Grp94 client proteins with Grp94 also involves a similarly rigorous matching of chaperone:client recognition surfaces. The mechanism by which this specific interaction might occur would be difficult to envision if the Grp94 client pool was as large and diverse as that of Hsp90. However, Grp94 has only a small set of client proteins, many of which fall into families that are structurally related, and has only two known cochaperones, CNPY3 and MZB1. The rigorous requirement for authentic Grp94 surfaces before Grp94 client protein maturation can occur may reflect a limited number of modes of client-chaperone interaction for this paralog.
